# A Novel Just-in-Time Contextual Mobile App Intervention to Reduce Sodium Intake in Hypertension: Protocol and Rationale for a Randomized Controlled Trial (LowSalt4Life Trial)

**DOI:** 10.2196/11282

**Published:** 2018-12-07

**Authors:** Michael P Dorsch, Lawrence C An, Scott L Hummel

**Affiliations:** 1 Department of Clinical Pharmacy College of Pharmacy University of Michigan Ann Arbor, MI United States; 2 University of Michigan Medical School Ann Arbor, MI United States

**Keywords:** geofencing, hypertension, mobile phone, sodium intake

## Abstract

**Background:**

High sodium intake is a significant public health problem in the United States. Interventions that lower sodium intake can decrease blood pressure and improve cardiovascular outcomes. Restaurants and grocery stores are prime targets for intervention with about 77% of all sodium intake in the average US diet coming from processed and restaurant foods.

**Objective:**

This study proposes that a mobile app intervention that promotes low-sodium alternatives at grocery stores and restaurants will reduce dietary intake of sodium and improve confidence following a low-sodium diet in hypertension.

**Methods:**

In this single-center, prospective, open-label study, patients will be randomized to a mobile app or usual care for 8 weeks. We will randomize 50 patients (age>18 years) diagnosed with hypertension and on antihypertensive therapy for at least 3 months in a 1:1 manner stratified by gender. Study subjects will receive the mobile app, LowSalt4Life, or usual dietary advice for 8 weeks. LowSalt4Life provides a multifaceted intervention based on just-in-time contextual tailored messages at grocery stores and restaurants. The primary endpoint is the change in the estimated 24-hour urinary excretion of sodium from spot urine. Secondary outcomes include change in the sodium content of the food frequency questionnaire, confidence in following a low-sodium diet, urine chloride and creatinine dipsticks, and blood pressure.

**Results:**

The project was funded in May 2016 until April 2018. This trial is currently enrolling patients. To date, 26 of the 50 patients needed have been enrolled. Results will be available in the Spring of 2019.

**Conclusions:**

This randomized controlled trial will test the efficacy of just-in-time contextual tailored messages through a novel mobile app 8-week intervention on urinary sodium excretion in patients with hypertension. We will address a critical evidence gap in the care of patients with hypertension. If effective, this intervention could be scaled to assess effects on blood pressure and cardiovascular events in hypertension.

**Trial Registration:**

ClinicalTrials.gov NCT03099343; https://clinicaltrials.gov/ct2/show/NCT03099343 (Archived by WebCite at http://www.webcitation.org/735HNzKlQ)

**International Registered Report Identifier (IRRID):**

PRR1-10.2196/11282

## Introduction

### Prevalence and Consequences of High Dietary Sodium Intake

High sodium intake is a significant public health problem in the United States. In a meta-analysis of 177,025 patients, higher sodium consumption was associated with higher risk of stroke (relative risk, RR, 1.23, 95% CI 1.06-1.43) and a trend toward higher cardiovascular risk (RR 1.14, 95% CI 0.99-1.32) [1]. Based on 2005 estimates, high dietary sodium is responsible for 102,000 deaths annually. The current federal guidelines advocate a daily sodium intake of <2300 mg with a further reduction to 1500 mg in persons aged ≥51 years and those of any age who are African American or have hypertension, diabetes, or chronic kidney disease (CKD), while the average sodium intake for all Americans aged ≥2 years is approximately 3400 mg/day [2].

### Effects of Interventions to Lower Sodium Intake

Interventions that lower sodium intake can decrease blood pressure and improve cardiovascular outcomes. The sodium-restricted Dietary Approach to Stop Hypertension eating plan reduced the systolic blood pressure (SBP) by 7.1 mm Hg in adults without hypertension and 11.5 mm Hg in adults with hypertension [3]. In an analysis of the Trials of Hypertension Prevention, participants randomized to low-sodium interventions had a 25% lower long-term risk of cardiovascular disease (RR 0.75, 95% CI 0.57-0.99) [4]. Following the 2003 introduction of a national salt reduction program in England, the 2011 national Health Survey estimated that the population’s sodium intake decreased by ~550 mg/day [5]. During that same timeframe, the population’s SBP declined by 3 (SD 0.3)/1.4 (SD 0.2) mm Hg, which was accompanied by a 42% decrease in stroke mortality (*P*<.001) and a 40% reduction in mortality due to ischemic heart disease (*P*<.001). After extensive adjustment for other potential factors, dietary sodium reduction was identified as the most likely contributor.

From a policy perspective in the United States, reducing sodium intake by 1200 mg/day is projected to prevent 60,000-120,000 coronary heart disease events, 32,000-66,000 strokes, 54,000-99,000 myocardial infarctions, and 44,000-99,000 deaths from any cause on an annual basis [6]. This reduction in sodium intake would also lead to US $10-24 billion savings in the health care system.

### Patients Need Assistance at Grocery Stores and Restaurants

Over half of Americans consume 1-3 restaurant meals per week, and 23% consume ≥4 restaurant meals per week. In addition, the use of prepackaged foods in home meal preparation has increased substantially in recent years. Restaurants and grocery stores are prime targets for interventions, with about 77% of all sodium intake in the average US diet coming from processed and restaurant foods [7,8]. In a recent study on consumer knowledge of sodium intake and food labeling, only half of the over 400 grocery store shoppers could accurately use sodium label information to choose low-sodium food options [9]. The American Heart Association guideline for the dietary approach to prevent and treat hypertension states, “any meaningful strategy to reduce salt intake must involve efforts of food manufacturers and restaurants” [10].

A qualitative study using a focus group that included patients with heart failure proposed 3 primary themes for nonadherence to low-sodium diet as follows: lack of knowledge, interference with socialization, and lack of food selections [11]. Many participants in this study felt they needed more detailed information about a low-sodium diet. They also expressed frustration in eating out at restaurants stating eating out was an obstacle to adhering to their prescribed diet. Finally, patients stated there are limited choices and poor taste of foods with low-sodium content.

### Advancements in Information Technology to Support Reduced Sodium Intake

We are currently witnessing broad social changes in the ways individuals expect to find and use information about their health. According to the Pew Research Center’s Internet and American Life Project, nearly 90% of households now have at least basic internet access, and 70% of households have broadband service [12]. The use of mobile phones has nearly become ubiquitous (with 90% of individuals owning a mobile phone), with a growing majority of individuals (65%) using smartphones [13]. We believe that a mobile app that provides location-based, tailored messages can reduce the dietary sodium intake and increase participants’ confidence in following a low-sodium diet.

The current state for self-monitoring of sodium intake in health information technology (IT) is to have users log all daily dietary intake, a process that requires substantial time and persistence. Patients or the mobile app then calculates dietary sodium intake and reviews it periodically, after the intake has already occurred. Our more proactive approach provides immediately available information on appropriate low-sodium options, as well as the ability to self-monitor the intake of specific high-sodium foods. This type of methodology could provide a significant change in thinking about self-monitoring for diets and nutrition.

From a health IT perspective, the use of contextual geofence-based push notifications is novel. This research will inform how users perceive these messages, which messages they understand most and ultimately if they act on the recommendations of the message. Furthermore, we will appreciate whether patients will accept that a mobile app follows their location to provide dietary recommendations. The research will inform the health IT community how to begin using location-based notifications in a way that does not turn users off to the idea of being tracked by someone.

**Table 1:** Conceptual model for health information technology intervention based on patients’ beliefs.

Patient beliefs about a low-sodium diet and theoretical framework		Intervention
**Lack of knowledge**		
	Attitude	Just-in-time messages based on geofencing technology when a patient enters a restaurant or grocery store provide the knowledge the patient needs to make an informed decision.
	Value proposition in food choices	Associating hypertension and blood pressure to lower sodium intake.
	Self-regulation	Patients will monitor the top 5 high-sodium foods.
**Interference with socialization**		
	Normative belief	Nonobtrusive food options are presented as a push notification when the patient enters a restaurant or scans an item at the grocery store.
**Lack of food selection**		
	Perceived control; Substitution and limitation as a food choice strategy	Several food options are presented that are usual food options at the restaurant and the ability to scan foods at the grocery store. This puts a patient in control of limiting sodium and substituting other low-sodium foods.

### Theoretical Framework for the Health Information Technology Intervention

The Theory of Planned Behavior, self-regulation, and mindful decision making are the theories used in the intervention to illicit a behavioral change. The direct determinants of behavioral intention are attitude, normative belief, and perceived control. The Theory of Planned Behavior suggests that the determinant of persons’ behavior is their behavioral intent [14]. Patients who hold strong beliefs that a positive outcome will result from a behavior will also have a positive attitude about that behavior. Normative beliefs are heavily dependent on the individuals’ motivation to comply with those around them. Patients also have to perceive control of the situation, making the behavior decision easy to perform.

In our intervention, patients planning what they are going to order at a restaurant will receive a push notification from the mobile app informing them of the low-sodium options at that restaurant. In food selection, the choice has to be within an area individuals perceive as normal. Providing a food choice on a restaurant menu that others would also purchase gives patients a feeling like they are complying with social norms. In addition, patients have to perceive control of the situation, making the behavioral decision easy to perform. In the setting of a push message provided by the mobile app, we present patients with several options to give them control over which food to choose.

The theory of self-regulation suggests that individuals striving to achieve a behavioral goal learn from their successes and failures. This knowledge is then used to develop strategies that can lead to goal attainment. In this theory, self-monitoring refers to individuals’ awareness of their engagement in a targeted behavior. An important factor in self-regulation is self-efficacy [15]. Self-efficacy refers to an individual’s belief in his or her ability to perform a specific behavior [16]. Accurate self-monitoring, feedback, and self-efficacy are essential components of the self-regulation cycle and are critical for lowering sodium intake [15,17-19].

Behavioral change interventions that focus on self-regulation are particularly well suited for automation. Through the use of the intervention, we will assess patients’ top 5 high sodium-containing food selections. These food selections will be embedded in the mobile app, and a daily push notification will prompt patients to document if they have reduced those foods from their diet.

Mindful decision making is key for patients to make positive food choices. Food choice decisions are complex and incorporate a variety of food behaviors [20]. Value negotiations are a component of food choice decisions. People negotiate competing values, prioritizing values to simplify food choice decisions. For example, patients with hypertension may value their health over other values like taste, cost, convenience, and relationships. Limitation and substitution are common strategies in food choice. Some may limit particular foods or ingredients to meet a goal, while others substitute one food for another to meet the same goal. Health IT can provide a behavioral change intervention for food choices. We provide just-in-time messages at a restaurant and the ability to scan items at a grocery store to promote limitation and substitution strategies. These strategies facilitate food choice decisions by making them automated, so values are not necessary for every situation.

### Aim and Hypothesis

The LowSalt4Life Trial will study the effectiveness of a newly developed mobile app that provides mobile phone sensor-based contextual push messages related to following a low-sodium diet. We will randomize patients with hypertension to the mobile app or standard of care for 8 weeks. From baseline to 8-week follow-up, we will assess the impact of the mobile app on the dietary intake of sodium (measured by 24-hour dietary recall and 24-hour urinary sodium excretion) and confidence following a low-sodium diet, measured by the Self-Care Confidence in Following a Low-Sodium Diet Scale (SCFLDS). We hypothesize that the group randomized to the mobile app will demonstrate improvement in these measurements compared with the control group (Table 1).

## Methods

### Trial Registration and Funding

This trial is registered at ClinicalTrials.gov (NCT03099343, received March 28, 2017) and is funded by the Agency for Healthcare Research and Quality (R21 HS024567). The authors are solely responsible for the design and conduct of this study, all study analyses, drafting and editing of the manuscript, and its final contents.

### General Design

This is a single-center, prospective, open-label designed clinical trial. Recruitment is being performed at the University of Michigan Health System through our patient recruitment platform and recruitment letters to patients that meet our study criteria. Enrolled patients will be randomized to the mobile app or usual care in a 1:1 fashion using the University of Michigan Consulting for Statistics, Computing and Analytics Research randomization tool. Patients randomized to the mobile app will receive a 30-minute training session about the mobile app and be instructed to use the mobile app for 8 weeks. To address the research bias in this unblinded trial, all primary outcomes measured are objective. Some outcomes are not objective and represent a limitation of this research. This study was approved by the University of Michigan Institutional Review Board.

### Study Subjects

We will enroll 50 patients aged >18 years diagnosed with hypertension and on antihypertensive therapy for at least 3 months. Patients will be excluded if they have CKD, heart failure, SBP >180 mm Hg, diastolic blood pressure >110 mm Hg, insulin-requiring diabetes mellitus, or are taking a loop diuretic, corticosteroid, or nonsteroidal anti-inflammatory medication. CKD is defined as known kidney damage (structural or functional abnormalities) or estimated glomerular filtration rate of <60 mL/min/1.73 m^2^ (CKD stage 3, 4, or 5). Initially consented participants will complete the Block Food Frequency Questionnaire (NutritionQuest) [21], and those with the estimated baseline sodium intake <2300 mg/day will be excluded before randomization.

### Intervention

The intervention with the mobile app includes the following 2 parts: (1) just-in-time contextual tailored messages that promote behavioral changes when a patient enters a grocery store and restaurant and (2) the ability to easily scan and search for the foods at grocery stores and restaurants to find options containing lower sodium content.

Just-in-time contextual tailored messages are generated on the basis of a multifaceted system. Initially, participants complete the Block Sodium Screener (NutritionQuest) and survey to assess their confidence in following a low-sodium diet. Users then create alternatives to the top 5 high sodium-containing foods and map these items to locations. In order to properly target messages, we use artificial intelligence algorithms that analyze the changes in mobile phones sensors (including Wi-Fi, Bluetooth, accelerometer, gyroscope, magnetometer, global positioning system). This takes each user’s past, recognizes his or her present context, and predicts future activity. These predictions then alert the mobile app that the user is entering a grocery store or restaurant. Once the mobile app knows the user’s context, the user receives a tailored message based on the context (grocery store or restaurant), their individual top 5 high sodium-containing foods (from the Block Sodium Screener) and their confidence following a low-sodium diet. These messages are delivered by push notifications.

**Figure 1:**
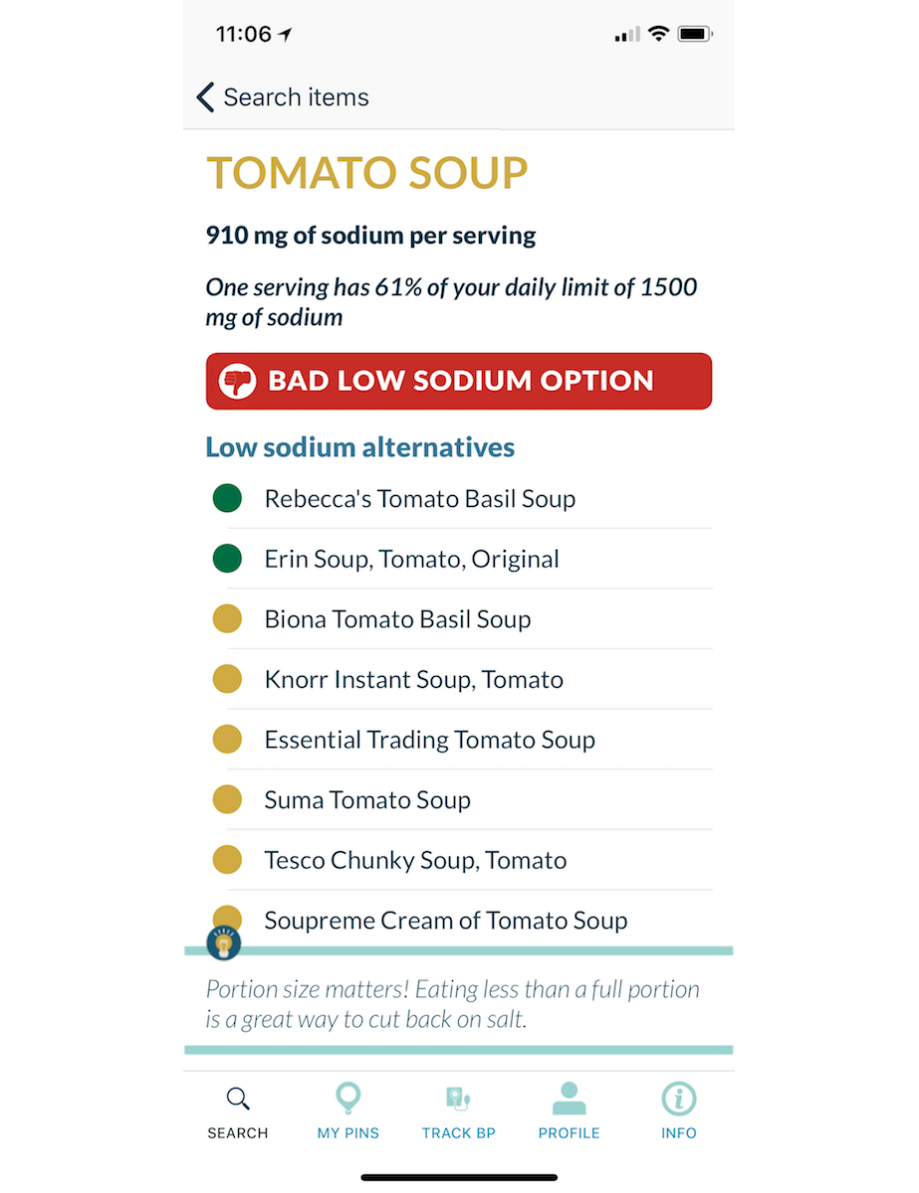
An example of the grocery store result screen for tomato soup using a traffic light signal.

When a user taps on a just-in-time contextual tailored message, the app is opened to the appropriate section for their context, grocery store or restaurant. The grocery store section provides users with the ability to scan Universal Product Codes on food packaging or text search at the grocery store. The mobile app provides feedback on the sodium content of the food using a traffic light signal red (≥480 mg/serving), yellow (≥120 mg/serving to <480 mg/serving), and green (<120 mg/serving) to show consumers, at a glance, whether a product is high, medium, or low in sodium [22-24]. In addition, the app will list lower sodium options in the same food category (Figure 1). In restaurants, users are presented 3 low-sodium meals for that specific restaurant that were curated by the investigators and be able to search the restaurant’s menu with items ordered by the sodium content, lowest to highest.

### Outcomes

**Figure 2:**
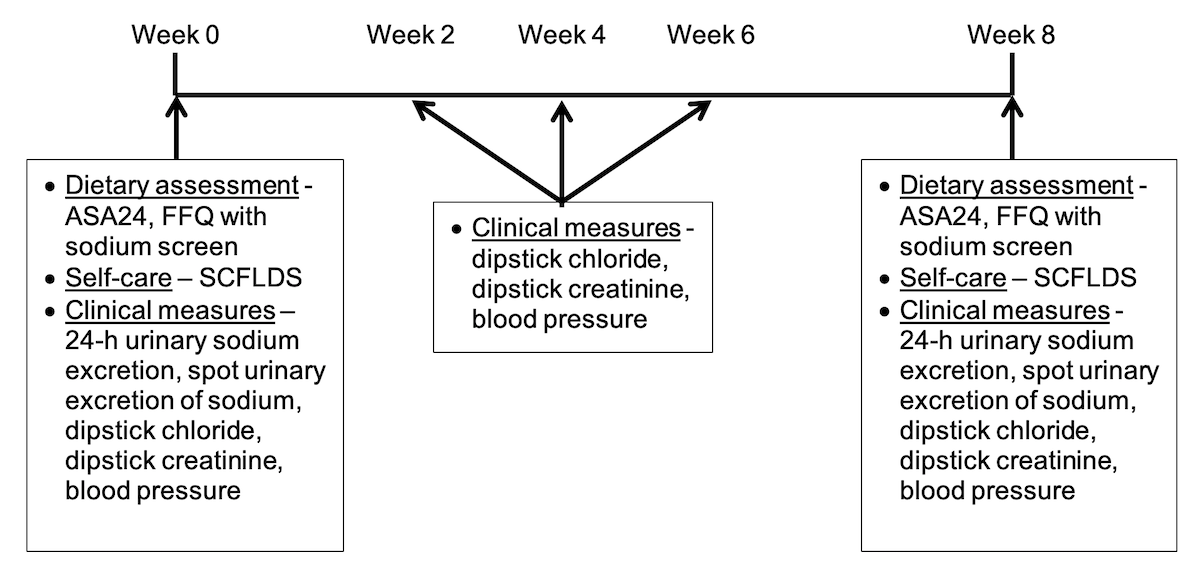
Summary of outcomes in the trial. ASA24: Automated Self-administered 24-Hour Dietary Recall; FFQ: Food Frequency Questionnaire; SCFLDS: Self-Care Confidence in Following a Low-Sodium Diet Scale.

The Automated Self-Administered 24-Hour Dietary Recall (ASA24), developed by the National Cancer Institute for dietary research, will be used to estimate the dietary sodium intake during the trial. The ASA24 is an electronic 24-hour recall website that allows patients to self-administer the survey in a user-friendly manner. The ASA24 allows researchers to send the survey at defined intervals and automatically performs a dietary analysis by converting the dietary recall data into nutrient information based on the Food and Nutrient Database for Dietary Studies. In order to provide a detailed interview, the website includes a meal-based quick list, meal gap review, review of forgotten foods, a final review, and a question about whether the day’s intake was usual or not. In this study, we will obtain the sodium content per day from the Food and Nutrient Database for Dietary Studies-based sodium content analysis completed by the ASA24 website. The ASA24 will be administered at week 0 and 8 of the study (see Figure 2 for details of outcomes).

The most widely used method for monitoring the sodium intake is urinary sodium excretion measured by a 24-hour urine collection. Patients will be instructed to collect all urine voids for 24 hours and return them for analysis for sodium excretion. In addition, the 24-hour urine excretion will be collected at baseline and after 8 weeks. This method is not convenient (eg, difficult to perform collections at work), and some participants may have incomplete urine collections. Owing to these challenges, we will also explore other methods to estimate sodium excretion that would be more practical for use in larger trials.

In addition to the gold standard, the first method to estimate the 24-hour urinary sodium excretion is spot morning urine excretion of sodium. The Kawasaki formula will be used to estimate the 24-hour sodium urinary excretion from a fasting morning urine sample [25]. This approach has been proven to provide a valid estimate of the sodium intake in several patient populations and in large-scale epidemiological studies [26]. Participants will be instructed to fast overnight, void in the morning, and provide us with a second morning urine sample on the day of the assessment. This measurement will be assessed at baseline and after 8 weeks of the study.

The second method for estimating the 24-hour urinary sodium excretion is with a spot urine sample using chloride and creatinine dipsticks. The dipstick method has been validated in patients with hypertension and correlates well with the 24-hour urinary sodium excretion (*r*=.86) [27]. The chloride dipstick is used as a surrogate for sodium excretion. The Quantab chloride dipstick (Hach, Loveland, CO, USA) is placed in a container filled less than halfway with urine. The chloride concentration is determined by observation of the change in color from brown to pale on the dipstick, which is the formation of silver chloride. The creatinine dipstick is similar to the chloride dipstick. The MultistixPro-10LS (Bayer, Elkhart, IN, USA) is a dipstick that includes a color pad for creatinine after it is placed in a container filled with urine. The color pad turns from yellow to green to indicate the creatinine concentration. The predicted 24-hour urinary sodium excretion is calculated on the basis of the 24-hour creatinine concentration calculated using sex, weight, race, and age. This model has been validated and correlates highly (*r*=.93) with 24-hour measurements of creatinine [28]. This measurement will be performed at baseline and after weeks 2, 4, 6, and 8.

The SCFLDS will be used to measure patients’ confidence in following a low-sodium diet. It assesses patients’ confidence in the ability to select and prepare low-sodium foods [29]. The tool consists of 7 items with 4 response options per item. The items ask patients to rate their level of confidence reading food labels, choosing low-sodium food during shopping, choosing low-sodium foods at restaurants, cooking low-sodium foods, choosing low-sodium foods at relatives or friends homes, estimating how much sodium they eat each day, and substituting low-sodium foods for high-sodium foods. The 4 response options are assigned a score of 1-4 (1=not confident; 2=somewhat confident; 3=very confident; 4=extremely confident) and then added for each question. Possible scores range from 7 to 28, and the higher score indicates greater confidence. The SCFLDS will be administered at baseline and after week 8 of the study.

Mobile device analytics will be collected to determine the extent to which the mobile app was used. This outcome will be key in determining the frequency and intensity of the intervention. After a participant receives a push notification on entry to a grocery store and restaurant, we will provide them with a push notification on exit asking them if they chose any of our alternative options. We will also collect any scanned items to understand better the specific foods that participants are seeking more information about. Blood pressure will be monitored on a weekly basis by patients, and the results will be entered into the mobile app and graphically represented.

### Statistical Approach

The primary endpoint this study is powered for is the estimated 24-hour urinary excretion of sodium from spot urine. Based on the data in patients with hypertension, we conservatively expect the 24-hour urinary excretion of sodium to decrease from 3400 (SD 1200) mg/24 hours to 2400 (SD 1200) mg/24 hours (35% reduction in sodium intake) in the mobile app group and a decrease from 3400 (SD 1200) mg/24 hours to 3300 (SD 1200) mg/24 hours in the usual care group [30]. The total sample size of 24 patients (12 in each group) and 32 patients (16 in each group) will provide 80% and 90% power, respectively, for a 35% reduction in sodium intake. In order to account for patients dropping out or incomplete follow-up, we are enrolling a total sample of 50 patients (25 in each group). We will use a two-sided *t* test to compare the change in each outcome over time in the mobile app group versus the usual care group. Other quantitative measures of sodium intake will be 24-hour urinary excretion of sodium and estimated 24-hour urinary excretion of sodium from chloride and creatinine dipsticks, which will be analyzed using a two-sided *t* test and repeated measures analysis of variance, respectively.

Although not powered to determine the impact, other measures of the effectiveness of this intervention will be summarized and evaluated. The percent change in confidence following a low-sodium diet will be analyzed using a two-sided *t* test. The change in blood pressure over time will be analyzed using repeated measures analysis of variance.

### Data Safety and Monitoring

A Data Safety and Monitoring Plan has been developed for this small intervention trial. Adverse events will be recorded from the day of the first study-related contact and reported to the Institutional Review Board. All participants who experience an adverse event will be referred for treatment. No formal stopping rule for harm has been established for this pilot study, as it evaluates standard-of-care treatment in a low-risk population.

## Results

The project was funded in 2016 and enrollment is expected to be complete in the Spring of 2019. The first results are expected to be submitted for publication in 2019.

## Discussion

The purpose of this randomized controlled trial is to test the efficacy of just-in-time contextual tailored messages using a mobile app for 8 weeks in patients with hypertension. This is a novel, first of a kind patient-centered decision support tool using geofencing technologies. We hypothesize that this intervention will improve sodium intake compared with the control group. By testing this novel approach, we could be developing a highly scalable mobile health model to help patients optimize their dietary habits in grocery stores and restaurants. If effective, the broader implications to other cardiovascular diseases and clinical outcomes should be explored.

## Abbreviations

ASA24: Automated Self-administered 24-Hour Dietary Recall
CKD: chronic kidney disease
IT: information technology
RR: relative risk
SBP: systolic blood pressure
SCFLDS: Self-care Confidence in Following a Low-sodium Diet Scale

## Appendix

Multimedia Appendix 1 Peer-review report from the Agency for Healthcare Research and Quality.
