# Supplementary material for: A Novel Just-in-Time Contextual Mobile App Intervention to Reduce Sodium Intake in Hypertension: Protocol and Rationale for a Randomized Controlled Trial (LowSalt4Life Trial)
Source: JMIR Res Protoc. 2018 Dec 7;7(12):e11282. doi: 10.2196/11282 (PMC6303672; doi:10.2196/11282)
Supplement: Multimedia Appendix 1 [file resprot_v7i11e11282_app1.pdf]

PROGRAM CONTACT: | **SUMMARY STATEMENT** (Privileged Communication) | *Release Date:*

Application Number: **1 R21 HS024567-01**

Principal Investigator

DORSCH, MICHAEL

Applicant Organization: UNIVERSITY OF MICHIGAN

Project Title: **A geofencing based adaptive messaging system to support patient self-management of a low sodium diet in hypertension**

SRG Action: **Impact Score: 36 Percentile: 17**

Human Subjects: 30-Human subjects involved - Certified, no SRG concerns
Animal Subjects: 10-No live vertebrate animals involved for competing appl.
Gender: 1A-Both genders, scientifically acceptable
Minority: 1A-Minorities and non-minorities, scientifically acceptable
Clinical Research - not NIH-defined Phase III Trial

Project Year	Direct Costs Requested	Estimated Total Cost
1	96,774	150,000
2	96,774	150,000
TOTAL	193,548	300,000

ADMINISTRATIVE BUDGET NOTE: The budget shown is the requested budget and has not been adjusted to reflect any recommendations made by reviewers. If an award is planned, the costs will be calculated by Agency grants management staff based on the recommendations outlined below in the COMMITTEE BUDGET RECOMMENDATIONS section.

RESUME AND SUMMARY OF DISCUSSION: This R21 health services research grant application from Dr. Michael Dorsch, from the University of Michigan, in Ann Arbor, MI, proposes to develop a mobile app that incorporates geofencing technology to provide just-in-time mobile messages for hypertensive patients to reduce their sodium intake. The research team will conduct clinical trial to evaluate the effectiveness of the mobile application in helping patients reduce sodium intake. The reviewers agreed that the concept of proactively trying to impact individual's food choices instead of having the application be purely reactive can make an impact in how useful the tool is to users. This method, based on the theory of planned behavior, is also completely automated and thus promises to be cost effective, an important consideration at the time when healthcare costs are rising. The reviewers added that linking the proposed project with the Factual location platform can provide accurate data on the locations of restaurants and grocery stores and linking it with the Nutritionix database can provide nutrition information on many grocery store items. The use of contextual geofence-based push notifications is novel. All of the research team is well experienced in their fields and seem to be a logical fit for this project. However, the reviewers also noted that the roles of the Principal and Co-investigators are not clearly described. There was no information on previous collaboration between the team members and no letters of support. In addition, there is little discussion about the usability of the proposed tool and the application does not identify mechanisms to ensure its proper utilization during the trial. Overall, the reviewers recommended this application for further consideration with an excellent to very good level of enthusiasm.

DESCRIPTION (provided by applicant): High dietary sodium intake is linked to incident hypertension, stroke, heart failure and kidney disease. U.S. federal guidelines advocate daily sodium intake of less than 2,300 milligrams (mg) with further reduction to 1,500 mg in persons who are 51 and older and those of any age who are African American and/or have hypertension, diabetes, or chronic kidney disease. The estimated average sodium intake for Americans is 3,400 mg per day. In recent years, consumption of pre-processed and restaurant foods has substantially increased, and more than 75% of sodium in the average U.S. diet now comes from these sources. Most patients prescribed a low sodium diet either do not understand or possess the information about sodium content of the foods they eat and current IT approaches to reduce sodium intake focus solely on counting the amount of dietary sodium eaten. We will develop a mobile application that incorporates geofencing technology and provides just-in-time mobile push messages at the 2 main locations hypertensive patients interact with processed and prepared foods, at a grocery store or eating at a restaurant. Geofencing technology allows the mobile application to be aware of the user's location and provide location-specific messages, vital for the just-in-time nature of the intervention. The proposed project will establish a geofencing based adaptive notification message system for the mobile application using participant feedback to facilitate reducing dietary sodium intake and will then determine the effectiveness of the mobile application in helping hypertensive patients reduce dietary sodium intake and feel more confident in following a low sodium diet. These data will be crucial for a planned randomized clinical trial to test the sustained effects of the mobile application on blood pressure and sodium intake. The application of this technological approach has positive implications in improving self-management and reducing disease morbidity.

PUBLIC HEALTH RELEVANCE: High dietary sodium intake is linked to high blood pressure, stroke, heart failure and kidney disease. U.S. federal guidelines advocate daily sodium intake of less than 2,300 milligrams (mg). The estimated average sodium intake for Americans is 3,400 mg per day. In recent years, consumption of pre-processed and restaurant foods has substantially increased, and more than 75% of sodium in the average U.S. diet now comes from these sources. We plan to develop and test the effectiveness of a mobile application to help hypertensive patients reduce the amount of dietary sodium intake using mobile push notifications at grocery stores and restaurants.

CRITIQUE NOTE: The sections that follow are the essentially unedited, verbatim comments of the individual committee members assigned to review this application. The attached commentaries may not necessarily reflect the position of the reviewers at the close of group discussion, nor the final majority opinion of the group. The above RESUME/SUMMARY OF DISCUSSION represents the evaluation of the application by the entire committee.

CRITIQUE 1

Significance:	1
Investigator(s):	2
Innovation:	2
Approach:	1
Environment:	1

Overall Impact:
Strengths
- This application proposes a study that addresses an important problem – excessive salt intake in patients with hypertension. It is using an innovative method (customized telephone messaging) that has been shown to be effective in other areas of medicine. This method is also completely automated and thus promises to be cost effective – an important consideration at the time when healthcare costs are rising. The proposal is well thought through and clearly written.

Weaknesses
- No weaknesses noted.

1. Significance:
Strengths
- Addresses an important medical problem – excessive salt intake in patients with hypertension.
- The health IT method proposed in the application holds significant promise – if successful, it could be used in a cost-effective fashion on a wide scale – a critical consideration for a condition as common as hypertension.

Weaknesses
- No weaknesses noted.

2. Investigators:
Strengths
- The Principal Investigator is a new PI to NIH. However, he has already held several other funding awards and has a number of publications to his name. Finally, thoughtfulness and clarify of the proposal itself inspire confidence in his ability to execute the study.

Weaknesses
- No weaknesses noted.

3. Innovation:
Strengths
- Telephone messaging is an innovative strategy that has already been shown to be successful in several medical realms. Its success is highly dependent on the content [of the messages] as well as on form [messages] through which this content is delivered. The investigators have clearly given a great deal of thought on how to make this content user-friendly and have high potential to succeed.
- Geofencing is another innovative health IT approach being used in this proposal. It allows for automation and customization of the messages directed to the patient, and may be the key to their success.

Weaknesses
- No weaknesses noted.

4. Approach:
Strengths
- The investigators' Research Plan carefully lays out the steps they will take to accomplish their Specific Aims. They start with hypotheses; conduct focus groups to develop these hypotheses further guided by patient feedback; and finally implement the resulting product and test its effectiveness. These are exactly the steps one needs to take when developing and evaluating a new IT technology.
- Outcomes being measured are appropriate.
- Proposed statistical analysis and power calculations are appropriate.

Weaknesses
- A single-blind (outcome analysis) design should be considered – there is nothing to prevent blinding the individual who will assess the outcomes to the intervention vs. control assignments.
- It could be of interest to evaluate on blood pressure as an exploratory outcome. But of course in a small trial of 4 weeks duration impact on blood pressure is less likely to be noticeable.

5. Environment:
Strengths
- The environment includes everything needed for success of the project: general research infrastructure (library, etc.), stimulating environment (multiple other investigators), strong technical / IT infrastructure available at the Center for Health Communications Research, as well as colleagues with the expertise needed for this project.

Weaknesses
- No weaknesses noted.

Protection of Human Subjects from Research Risks: Acceptable.

Inclusion of Women and Minority Subjects: Acceptable.

Inclusion of AHRQ Priority Populations: Acceptable.

Degree of Responsiveness: Acceptable.

Budget and Period of Support: Acceptable.

CRITIQUE 2

Significance:	4
Investigator(s):	3
Innovation:	5
Approach:	5
Environment:	2

Overall Impact:
Strengths
- Reducing sodium intake by making individual aware of their food choices and providing alternate options based in their location can make an impact on individual's choices.

- The concept of proactively trying to impact individual's food choices instead of having the application be purely reactive can make an impact in how useful the tool is to users.

Weaknesses

- One of the challenges in IT evaluation is ensuring that the users utilize the application. The proposal does not identify mechanisms for ensuring that users utilize the application during the trial or if they are not utilizing finding out why (i.e. a technical issue, a training issue, etc.).
- There is little discussion about the usability of the application. It would be hard to assess the usefulness of the application without addressing the usability of it.

1. Significance:
Strengths

- Identifying mechanisms to lower high sodium intake levels through the use of mobile applications is significant.
- The use of geofencing as a mechanism to identify where an individual is in order to help identify the types of food that may be lower in sodium content can make a significant impact.

Weaknesses

- The use of a randomized control trial without addressing usability issue could negatively impact the results.
- The system design process is not clear. Is this a user-centered design process? Are users only involved in the design process at the beginning?

2. Investigators:
Strengths

- The research team has a solid background in the problem space.

Weaknesses

- The research team should consider bringing on a usability/Human-Computer Interactions expert to oversee the design/evaluation of the system.

3. Innovation:
Strengths

- The use of geofencing to provide food recommendations is potentially innovative.

Weaknesses

- The design/data collection process is not innovative.
- The use traffic light labels for sodium levels are not innovative.

4. Approach:
Strengths

- The use of traffic light labels as a way to address literacy concerns is strength.
- The use of theory of planned behavior to guide the behavior change approach is a strength.

Weaknesses

- Little discussion of the design process or how users would be involved in the process.
- Little discussion about addressing usability issues.
- The focus of the application seems to be on the messaging and does not really attempt to address the technology challenges.

5. Environment:
Strengths

- There seems to be strong organizational support for the project.

Weaknesses

- No weaknesses noted.

Privacy and Security Protections in the Development and Implementation of Health IT System: Acceptable.

Strengths

- The proposal has a detailed description of how individual data will be protected.

Weaknesses

- No weaknesses noted.

Protection of Human Subjects from Research Risks: Acceptable.

Strengths

- The risks are minimal and the protection seems adequate.

Weaknesses

- No weaknesses noted.

Inclusion of Women and Minority Subjects: Acceptable.

Strengths

- Women and minority subjects will be recruited in the proportion to their population in the county where the recruiting will take place.

Weaknesses

- No weaknesses noted.

Inclusion of AHRQ Priority Populations: Acceptable. The proposal will focus on older adults.

Degree of Responsiveness: The proposal is responsive to the FOA

Budget and Period of Support: The budget and period of support appears appropriate.

CRITIQUE 3

Significance:	2
Investigator(s):	2
Innovation:	2
Approach:	4
Environment:	1

Overall Impact:

Strengths

- The current self-monitoring of sodium intake depends on users login in daily dietary intake after the intake has already occurred, just-in-time mobile messages can be more proactive. If successful this project can have positive implications in improving self-management and reducing disease morbidity.
- Counting the dietary sodium intake does not work most of the time, looking for an alternative through focusing on the top 5 high sodium containing food selections and recommending healthier alternative can be more acceptable by patients.
- Well-founded theoretical framework based on the theory of planned behavior.
- Linking the application with the Factual location platform can provide accurate data on the locations of restaurants and grocery stores and linking the application with Nutritionix database can provide nutrition information on many grocery store items.
- The research team plans to develop push notifications that will evoke a behavior change.

Weaknesses

- The Timeline needs to be more detailed.

- Patients may become concerned about privacy and security because the application uses GPS location services.
- It is not clear if inclusion/exclusion would take into account the smartphone data plans. There was no discussion on how to account for participant data usage.
- 4 weeks may not be long enough period to decide if the intervention is working. Patients could get alert fatigue and may no longer pay attention to messages. Especially, if occasionally these messages are triggered in error.
- The roles of the PI and Co-investigators are not clearly described.

1. Significance:
Strengths
- The current self-monitoring of sodium intake depends on users login in daily dietary intake after the intake has already occurred, just-in-time mobile messages can be more proactive.
- If successful this project can have positive implications in improving self-management and reducing disease morbidity.
- The use of contextual geofence-based push notifications is novel and can inform future health IT research on how users perceive such messages.

Weaknesses
- No weaknesses noted.

2. Investigators:
Strengths
- All of the research team is well experienced in their fields and seem to be a logical fit for this project.

Weaknesses
- The roles of the PI and Co-investigators are not clearly described.
- There was no information on previous collaboration between the team members.
- There were no letters of support attached to this application.

3. Innovation:
Strengths
- Developing mobile application that incorporates geofencing technology and providing just-in-time messages for hypertensive patients is innovative.
- Counting the dietary sodium intake does not work most of the time, looking for an alternative through focusing on the top 5 high sodium containing food selections and recommending healthier alternative can be more acceptable by patients.

Weaknesses
- No weaknesses noted.

4. Approach:
Strengths
- Mobile health can provide behavioral change intervention for food choices.
- Well-founded theoretical framework based on the theory of planned behavior.
- Linking the application with the Factual location platform can provide accurate data on the locations of restaurants and grocery stores.
- Linking the application with Nutritionix database can provide nutrition information on many grocery store items.
- The research team plans to develop push notifications that will evoke a behavior change.

Weaknesses

- 4 weeks may not be long enough period to decide if the intervention is working. Patients could get alert fatigue and may no longer pay attention to messages. Especially, if occasionally these messages are triggered in error.
- The focus on the sodium content in the type of food purchased (red, yellow, and green) without accounting for the quantity can be misleading.
- It is not clear how the application will decide on how to offer lower sodium options from within the same food category.
- The Timeline needs to be more detailed.
- Patients may become concerned about privacy and security because the application uses GPS location services.
- It is not clear if inclusion/exclusion would take into account the smartphone data plans. There was no discussion on how to account for participant data usage.

5. Environment:
Strengths

- University of Michigan environment is more than adequate to conduct this study.

Weaknesses

- No weaknesses noted.

Privacy and Security Protections in the Development and Implementation of Health IT System*:*
Strengths

- There is a section entitled "Plan for Privacy and Security Protections in the Development and Implementation of Health IT Systems" included in the Research Strategy section of the grant application. The applicant identified detailed plan to protect the Privacy and Security of the patients' protected health information (PHI).

Weaknesses

- Patients may become concerned about privacy and security because the application uses GPS location services.

Protection of Human Subjects from Research Risks: Acceptable.

Inclusion of Women and Minority Subjects: Acceptable.

Inclusion of AHRQ Priority Populations: Acceptable.

Degree of Responsiveness: Responsive.

Budget and Period of Support: It is not clear if there were funds allocated to pay for the cost of the focus groups. The budget may need to account for data plans of the smartphones. The role of the MCRU (services are $16,000 in year 2) is not clearly described.

THE FOLLOWING RESUME SECTIONS WERE PREPARED BY THE SCIENTIFIC REVIEW ADMINISTRATOR TO SUMMARIZE THE OUTCOME OF DISCUSSIONS OF THE REVIEW COMMITTEE ON THE FOLLOWING ISSUES:

PROTECTION OF HUMAN SUBJECTS (Resume): ACCEPTABLE.

INCLUSION OF WOMEN PLAN (Resume): ACCEPTABLE

INCLUSION OF MINORITIES PLAN (Resume): ACCEPTABLE

INCLUSION OF AHRQ PRIORITY POPULATIONS PLAN (Resume): ACCEPTABLE

COMMITTEE BUDGET RECOMMENDATIONS: The budget was recommended as requested
